# The susceptibility of disulfide bonds towards radiation damage may be explained by S⋯O interactions

**DOI:** 10.1107/S2052252520008520

**Published:** 2020-07-24

**Authors:** Rajasri Bhattacharyya, Jesmita Dhar, Shubhra Ghosh Dastidar, Pinak Chakrabarti, Manfred S. Weiss

**Affiliations:** a Department of Biochemistry, Bose Institute, P-1/12 CIT Scheme VIIM, Kolkata 700 054, India; b Division of Bioinformatics, Bose Institute, P-1/12 CIT Scheme VIIM, Kolkata 700 054, India; c Macromolecular Crystallography (HZB-MX), Helmholtz-Zentrum Berlin für Materialien und Energie, Albert-Einstein-Strasse 15, D-12489 Berlin, Germany

**Keywords:** radiation damage, disulfide bonds, S⋯O interactions, quantum-chemical calculations, NBO, electron transfer

## Abstract

Radiation-induced damage to protein crystals during X-ray diffraction data collection is a major impediment to obtaining accurate structural information on macromolecules. Some of the specific impairments that are inflicted upon highly brilliant X-ray irradiation are metal-ion reduction, disulfide-bond cleavage and a loss of the integrity of the carboxyl groups of acidic residues. With respect to disulfide-bond reduction, previous results have indicated that not all disulfide bridges are equally susceptible to damage. A careful analysis of the chemical environment of disulfide bonds in the structures of elastase, lysozyme, acetylcholinesterase and other proteins suggests that S—S bonds which engage in a close contact with a carbonyl O atom along the extension of the S—S bond vector are more susceptible to reduction than the others. Such an arrangement predisposes electron transfer to occur from the O atom to the disulfide bond, leading to its reduction. The interaction between a nucleophile and an electrophile, akin to hydrogen bonding, stabilizes protein structures, but it also provides a pathway of electron transfer to the S—S bond, leading to its reduction during exposure of the protein crystal to an intense X-ray beam. An otherwise stabilizing interaction can thus be the cause of destabilization under the condition of radiation exposure.

## Abstract 2

Irradiation of proteins with intense X-ray radiation generates specific structural and chemical alterations, such as metal-ion reduction, reduction of disulfide bonds and decarboxylation reactions. An analysis of a set of disulfide bonds in known structures suggests that the most susceptible disulfide bonds feature a carbonyl O atom positioned along the extension of the S—S bond vector, forming a stabilizing S⋯O interaction, which upon irradiation leads to polarization of the disulfide bond and its eventual reduction.

## 1. Introduction   

The advent of highly brilliant X-ray sources has made it possible for atomic resolution structures to be determined in macromolecular crystallography. However, the high doses of ionizing radiation also cause the crystalline order of the sample to be damaged, even at cryogenic temperatures. This can seriously degrade the quality of the data obtained, which may eventually lead to artifacts in the density map that defy biological interpretation (Garman, 2010 ▸). Radiation damage arising from photoelectric absorption and inelastic scattering can be nonspecific (Henderson, 1990 ▸; Nave, 1995 ▸; Teng & Moffat, 2000 ▸), leading to degradation of the crystal integrity, or specific, leading to metal-ion reduction, disulfide-bond reduction and concomitant cleavage and decarboxylation reactions (Helliwell, 1988 ▸; Burmeister, 2000 ▸; Ravelli & McSweeney, 2000 ▸; Weik *et al.*, 2000 ▸; Carugo & Carugo, 2005 ▸). The specific damage arises from the fact that photoelectrons and the radicals formed by the absorption of photons by the protein crystals do not spread stochastically over the whole crystal, but are preferentially trapped within specific groups in the protein molecule. Metal centers in redox enzymes are particularly sensitive. As a result, some oxidation states that are stable in horseradish peroxidase and nickel superoxide dismutase are difficult to characterize crystallographically as the oxidized forms are quickly reduced by photoelectrons (Carugo & Carugo, 2005 ▸). Next to metal-center reduction, it has also been shown that disulfide bonds are affected. After capturing a photoelectron, an S—S bond converts into an anionic radical intermediate (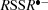). This intermediate can either capture a further electron, leading to complete reduction of the S—S bond, or revert back to the oxidized state (Close & Bernhard, 2019 ▸). It has been speculated that cryoprotectant molecules or other electron or radical scavengers may play a role in mediating this repair process (Carpentier *et al.*, 2010 ▸; Kauffmann *et al.*, 2006 ▸). It has been proposed that the formation of hydrogen gas inside the sample is mainly responsible for the loss of high-resolution information in diffraction experiments (Meents *et al.*, 2010 ▸).

Although some fragmentary ideas have emerged on the chemical modifications that arise on the irradiation of crystals, the underlying mechanisms are still not clear (Ravelli & McSweeney, 2000 ▸). For example, it has been observed that disulfide bonds in the same structure are affected differently by X-ray exposure (Weik *et al.*, 2000 ▸; Weiss *et al.*, 2005 ▸), although all attempts to relate specific geometric or chemical features of a disulfide bond to its susceptibility to reduction have not been very successful (Gerstel *et al.*, 2015 ▸). Disulfide bonds (Bhattacharyya *et al.*, 2004 ▸), and sulfur-containing residues in general (Pal & Chakrabarti, 1998 ▸, 2001 ▸), occur in various conformations, are found in varied chemical environments in protein structures and partake in different non­bonded interactions. Here, we report a possible connection between the susceptibility of disulfide bonds to reduction by radiation damage and the presence of a directional carbonyl O⋯S—S interaction in the structure. This provides an interesting scenario in which an otherwise stabilizing S⋯O interaction can destabilize the structure when exposed to intense X-ray radiation.

## 2. Materials and methods   

Six proteins containing disulfide bridges, elastase, hen egg-white lysozyme (HEWL), *Torpedo californica* acetylcholin­esterase (AChE), winged-bean chymotrypsin inhibitor (WCI), thaumatin and insulin, were used in this study. For elastase, 13 structures (named A-0 to A-12; Table 1 ▸) were generated from consecutive diffraction data sets collected from a single crystal on the XRD1 beamline at the ELETTRA synchrotron, Trieste, Italy at a wavelength of 1.00 Å (Weiss *et al.*, 2005 ▸). The data were reduced using *DENZO* (Otwinowski & Minor, 1997 ▸), *SCALA* and *TRUNCATE* (Winn *et al.*, 2011 ▸). The quality of the underlying data is excellent. In order to ensure maximum comparability, all data sets were truncated to a resolution of 1.85 Å [see Tables 1(*a*) and 1(*b*) in Weiss *et al.* (2005 ▸)]. With *I*/σ(*I*) values exceeding 40 in the outermost resolution shell and an overall Wilson *B* value of around 13 Å^2^, the true resolution of the data is close to 1.0 Å. The structure was refined using *REFMAC* (Murshudov *et al.*, 2011 ▸) to convergence against the first data set A-0 (*R* = 16.49%, *R*_free_ = 20.48%). The corresponding PDB code is 1uvo. For the other data sets A-1–A-12, the structure refined against the A-0 data was taken and refined for a few cycles and used without further modification. Since the other data sets A-1–A-12 are derived from the same crystal, the refined A-0 model can be considered to be a very good model for the remaining data sets. Based on our experience, a few cycles of further refinement are absolutely sufficient to bring all of the relevant bond-length and bond-angle parameters to their final position. For each of the individual data sets, the dose received per data set is estimated to be of the order of 0.15–0.2 MGy (Kmetko *et al.*, 2006 ▸). For the other proteins, the atomic coordinates used in the analysis were extracted from the Protein Data Bank (PDB; Berman *et al.*, 2000 ▸). The PDB codes (with resolution and *R* factor) are HEWL, 1qio (1.20 Å, 0.19; Weik *et al.*, 2000 ▸); AChE, 2wg2 (1.95 Å, 0.17; Sanson *et al.*, 2009 ▸); WCI, 4wbc (2.13 Å, 0.20; Ravichandran *et al.*, 1999 ▸); thaumatin, 5wr8 (1.55 Å, 0.13; Masuda *et al.*, 2017 ▸); insulin, 1zeh (1.50 Å, 0.16; Whittingham *et al.*, 1998 ▸).

We would like to define the susceptibility of a disulfide bond towards radiation-induced reduction qualitatively as the degree of appearance of difference electron-density peaks next to the S atoms. Upon partial reduction of an S—S bond, a disulfide radical anion is formed (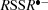), which is characterized by an elongated S—S bond length of about 2.8 Å (Weik *et al.*, 2002 ▸). Owing to the restraints for the S—S bond length used in the refinement procedure, electron-density difference map peaks will appear next to the S atoms. The higher these peaks are, the higher the degree of reduction.

**Figure 1:**
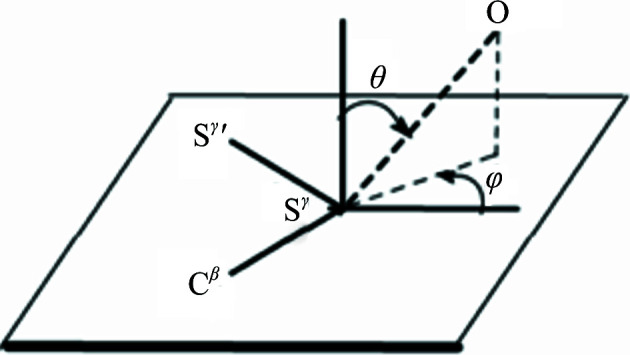
Spherical polar angles (θ, φ) defining the position of the carbonyl O atom relative to the disulfide plane. Of the two S atoms, that making the contact is labeled S^γ^ and the other is labeled S^γ^′. The figure is based on the convention used in Bhattacharyya *et al.* (2004 ▸).

The coordinate system used in the analysis is shown in Fig. 1 ▸. A cutoff distance of 4.0 Å was used to identify carbonyl O atoms which were in contact with any S atom of the disulfide bridge under consideration (Bhattacharyya *et al.*, 2004 ▸; Chakrabarti & Bhattacharyya, 2007 ▸). θ is the polar angle between the normal to the sulfide plane and the S^γ^⋯O vector (if θ > 90° then θ is made equal to 180° − θ, so that contacts above or below the plane are assumed to be equivalent; *i.e.* 0° < θ < 90°). φ is the azimuthal angle between the extension of the bisector of the angle C^β^—S^γ^—S^γ′^ and the projection of O in the disulfide plane.

**Figure 2:**
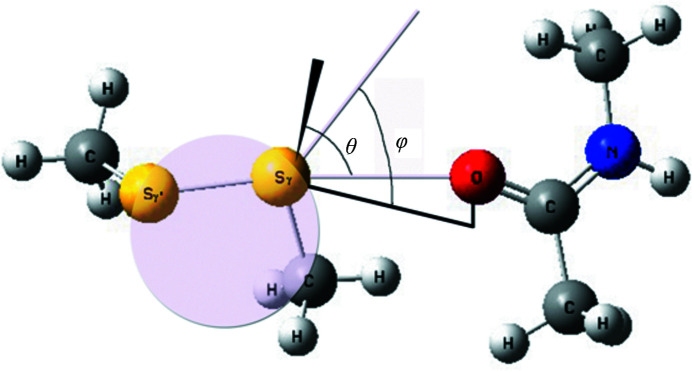
Model of CH_3_-S-S-CH_3_ interacting with CH_3_-CO-NH-CH_3_ at θ = 90°, φ = −60° and S⋯O distance = 3.08 Å. The spherical polar angles as defined in Fig. 1 ▸ are indicated relative to the normal to the plane and the bisector to the angle S^γ^′—S^γ^—C^β^.

**Figure 3:**
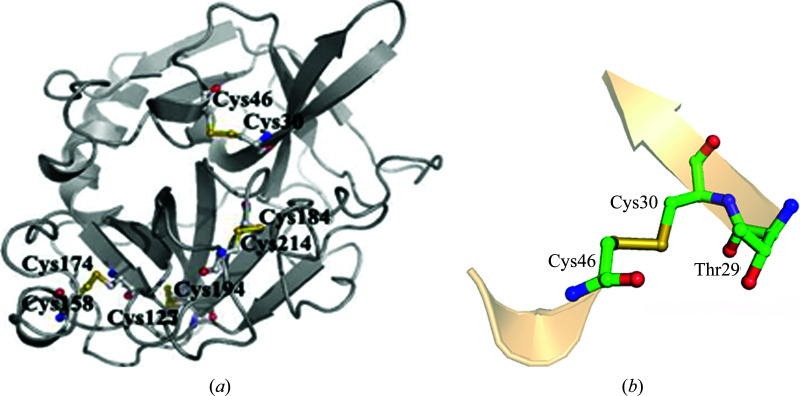
Ribbon diagram of elastase with a ball-and-stick representation of the disulfide bonds. All of the disulfide-bridged cysteine residues have zero relative solvent accessibilities. The detailed environment of the Cys30–Cys46 disulfide bond is shown in (*b*).

Energetics and charge transfer in S⋯O interactions were quantified using the ‘disulfide–amide’ model, consisting of two fragments, CH_3_-S-S-CH_3_ and CH_3_-CO-NH-CH_3_ (Fig. 2 ▸), constructed using *GaussView* 5.0.9 (Frisch *et al.*, 2013 ▸). It mimics the distance (3.08 Å) and relative orientation observed in one of the susceptible disulfide bonds (Cys30–Cys46) in elastase (PDB entry 1uo6; Mueller-Dieckmann *et al.*, 2004 ▸), which has a slightly different value to that (3.13 Å) in Table 2 ▸. All of the heavy atoms in the disulfide–amide model were made to coincide with the Cys30–Cys46 disulfide bond and the carbonyl of Thr29 [Fig. 3 ▸(*b*)]. As the two interacting groups are from neighboring residues, when the two fragments in the disulfide–amide model are made to superimpose on the corresponding groups in elastase, there is a steric clash involving methyl groups. To ameliorate the situation, the amide group was rotated by ∼90°, but maintaining the same S⋯O distance. This relates to the orientation corresponding to the combination of θ, φ values of (90°, −60°) (Fig. 2 ▸). The configurations corresponding to several other combinations of θ, φ, such as (90°, 0°), (90°, 50°), (45°, −60°), (45°, 0°), (45°, +50°) and (0°, −60°), were generated using *UCSF Chimera* (Pettersen *et al.*, 2004 ▸). It may be mentioned that we used a φ of +50° instead of +60°, as with the latter value S^γ^ and S^γ^′ become equidistant from the O atom. In addition, for the configuration shown in Fig. 2 ▸ we also rotated the amide moiety around the C=O axis in 30° increments and carried out energy and ESP (electrostatic potential) calculations. The energies of the configurations were measured using both Hartree–Fock and density functional theory (DFT) from single-point energy calculations. We used 6-31G++(2d,2p) as the basis set and the B3LYP functional for DFT. All quantum-mechanical calculations were performed using the *Gaussian* 09 package (Frisch *et al.*, 2013 ▸). Natural bond orbital (NBO) second-order perturbation analyses using both DFT and Hartree–Fock theory were also carried out to calculate stabilization energy, charge separation and orbital interactions. *NBO* version 3.1 as implemented in *Gaussian* 09 was used for this purpose (Glendening *et al.*, 1995 ▸). Molecular illustrations were made using *PyMOL* (DeLano, 2002 ▸), *MOLSCRIPT* (Kraulis, 1991 ▸) and *UCSF Chimera* (Pettersen *et al.*, 2004 ▸).

## 3. Results   

### 3.1. Elastase   

Elastase is a serine protease, the structure of which is stabilized by four disulfide bonds bridging cysteine residues 30 and 46, 127 and 194, 158 and 174, and 184 and 214. The decreasing order of susceptibility to radiation damage of the disulfide bonds, as inferred from the appearance of difference electron density as a function of increased dose, is Cys158–Cys174 > Cys30–Cys46 > Cys127–Cys194 > Cys184–Cys214, as was observed by Weiss *et al.* (2005 ▸). All four disulfide bonds are completely buried in the structure (Fig. 3 ▸), and as such the difference in their stability cannot be rationalized in terms of solvent exposure. Various bonded and nonbonded parameters involving the disulfide bonds were analyzed, looking for trends in data sets collected after different lengths of radiation exposure.

### 3.2. Bond parameters   

The S—S bond length may also be used as an indicator for reduction of the respective disulfide bond. The standard S—S bond length for an oxidized disulfide bond is 2.02 Å (Engh & Huber, 1991 ▸), whereas that for a partially reduced disulfide radical anion is about 2.8 Å (Weik *et al.*, 2002 ▸). Since crystallographic structures are averages over the whole crystal and the time that it takes to collect the underlying diffraction data, a gradual lengthening of the S—S bond length can be taken as evidence for the extent of reduction. However, there are two caveats. Firstly, this only works in the early stages of radiation damage when the degree of reduction is small, because the restraints used in structure refinement will keep the S—S bond length short. Secondly, as soon as the degree of reduction is high enough so that two distinct structures can be derived from the electron density (oxidized and reduced), the S—S bond length of the oxidized disulfide should revert back to the standard 2.02 Å.

Except for Cys184–Cys214, there is a tendency for the S—S bond length to increase with increasing exposure to radiation. The maximum increase of 0.06 Å is observed for the Cys30–Cys46 bond (Table 1 ▸). However, the most susceptible S—S bond, Cys158–Cys174, did not show any increase: the bond distance (2.07 Å) was on the longer side to start with and was comparable to the final value achieved (2.09 Å) by the second-most susceptible S—S bond (Cys30–Cys46).

Two angles centered on the S atom show some trends for all four disulfide bonds. The angle corresponding to the first half-cystine is larger than that for the second one [the average values for the angles in the first data set are 105.2 (±1.8)° and 101.4 (±2.3)°, respectively]. For the two susceptible disulfide bonds, the first angle increases (by ∼3°), while the second angle decreases, especially for the Cys30–Cys46 moiety (∼4°).

Considering χ_3_, there is no change in the conformation of the disulfide bonds. However, it may be pointed out that the last two comparatively stable disulfide bonds have positive χ_3_ torsion angles, while the angles are negative for the first two unstable disulfide bonds. The combined plot of χ_1_ and χ_2_ values also indicates that the points lie in the clustered regions shown in Figs. 2(*b*) and 2(*c*) in Bhattacharyya *et al.* (2004 ▸).

### 3.3. S⋯O interactions   

**Figure 4:**
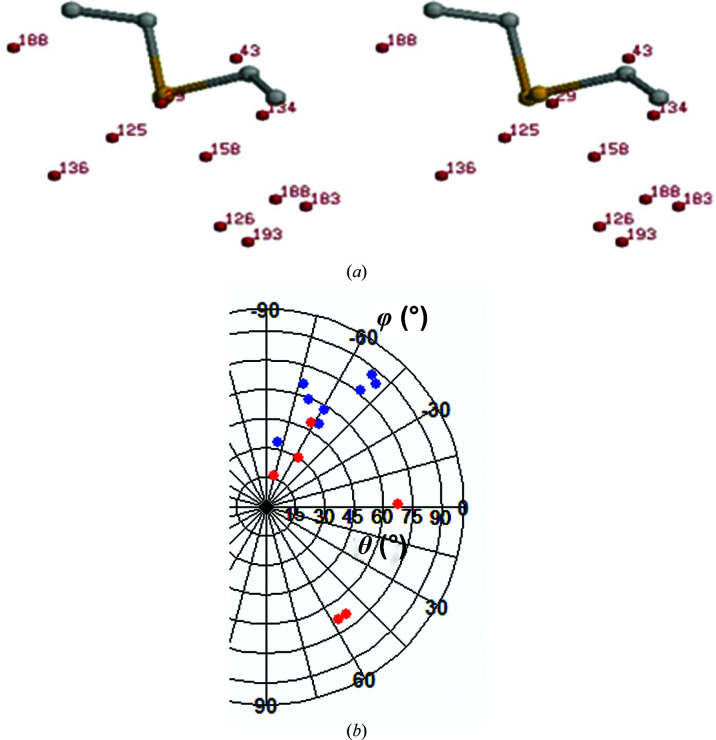
(*a*) Scatter plot (in stereo) of the distribution of carbonyl O atoms around the disulfide plane in elastase; the atoms interacting with all four disulfide bonds from the first data set (A-0) are shown. The C^β^—S^γ^—S^γ^′ plane (where S^γ^ is the atom in contact with O) is the common frame around which the coordinates of the O atoms are expressed; these are labeled with the numbers of the residues that they belong to (given in Table 2 ▸). (*b*) Polar graph of θ *versus* φ values taken from Tables 2 ▸, 4 ▸ and 5 ▸: those for the susceptible disulfide bonds are in blue (if there are multiple contacts, the first entry is used), and the less susceptible disulfide bonds are in red (that with the shortest contact distance is used if there are multiple entries).

In Table 2 ▸, the carbonyl O atoms interacting with the disulfide bonds and the associated parameters defined in Fig. 1 ▸ are given. The two unstable disulfide bonds each have a short S⋯O contact with a peptide O atom (3.24 and 3.13 Å, respectively, for the Cys158–Cys174 and Cys30–Cys46 disulfide bonds). Thus, the existence of a short contact may facilitate the disruption of the S—S bond. Additionally, the distribution of all of the interacting carbonyl O atoms around a common frame of the disulfide plane, shown in Fig. 4 ▸(*a*), indicates that the carbonyl O atoms of Cys158, Thr29 and Ala43, which make very close contact with an S atom of these sensitive disulfide bonds, are positioned along the extension of the S^γ^′—S^γ^ bond. This can also be seen from the data presented in Table 2 ▸. The S^γ^′—S^γ^—O angle is in the range 140–170° and the C^β^—S^γ^—O angle is <90° for these inter­actions, which is usually not the case for the remaining short contacts. The other set of parameters, θ and φ (Fig. 1 ▸), also gives the same indication. Values of ∼90° and −60°, respectively, indicate the location of the O atoms along the extension of the S^γ^′—S^γ^ bond. Although in solution the nucleophilicity of a carbonyl group of a small molecule is invariant of its surrounding in the molecule, the geometry imposed by the three-dimensional structure must be expected to modulate the nucleophilicity inside protein structures. Consequently, the three carbonyl groups with their position behind the S—S bond are poised to attack the disulfide bond when excited by photons. It seems possible that when the S⋯O interaction involves two atoms belonging to the same or neighboring residues (the first two entries in Table 2 ▸), the contact distance is rather constrained to be shorter, with optimally oriented molecular orbitals, making the disulfide bond more susceptible to damage.

The change in the S⋯O distance with exposure is considered next. The two susceptible disulfide bonds (Cys158–Cys174 and Cys30–Cys46) are involved in S⋯O contacts that increase (by 0.09 and 0.22 Å, respectively) on going from the first data set to the last (Table 3 ▸). The others, which have a longer contact distance to start with, do not have a comparable lengthening. Thus, with sustained X-ray exposure, as the disulfide bond breaks there is continued weakening of the S⋯O interaction.

### 3.4. S⋯O interactions in HEWL   

For hen egg-white lysozyme, the disulfide bond Cys6–Cys127 is the most susceptible, whereas Cys76–Cys94 is partially cleaved; the remaining two disulfide bonds, Cys30–Cys115 and Cys64–Cys80, are left rather unperturbed (Weik *et al.*, 2000 ▸). Although the most susceptible disulfide bond is relatively more exposed to the solvent, there is no distinction among the other three. The most susceptible disulfide bond, Cys6–Cys127, has a peptide O atom in contact at at distance of 3.40 Å, whereas the distance is longer for all other disulfide bonds (Table 4 ▸). The linearity of the interaction, as given by the S^γ^′—S^γ^—O angle, is also the maximum for this interaction. This is corroborated by the C^β^—S^γ^—O angle being acute. The disposition of the carbonyl O atom along the extension of the S—S bond is also indicated by the values of the two spherical polar angles, θ and φ, which are similar to the two most susceptible disulfide bonds in elastase. The spherical polar angles for the O atoms in contact with S for all of the disulfide bonds used in the analysis, susceptible and non-susceptible, indicate fairly distinct clustering for the two groups of points, especially the former [Fig. 4 ▸(*b*)].

### 3.5. S⋯O interactions in AChE   

*T. californica* acetylcholinesterase (AChE), with a subunit molecular weight of 65 000 Da, contains three intra-chain disulfide bonds. Of these, the Cys254–Cys265 disulfide bond disintegrates during the course of exposure, whereas the other two, Cys67–Cys94 and Cys402–Cys521, appear to be substantially more stable over the same X-ray dose (Weik *et al.*, 2000 ▸). Here, the susceptible disulfide bond has a peptide O atom at a slightly longer distance (3.68 Å) than those observed for stable disulfide bonds, but this O atom is situated at the posterior extension of the S^γ^—S^γ^′ bond, with an S^γ^′—S^γ^—O angle of 146° and a C^β^—S^γ^—O angle of 65° (Table 4 ▸). The θ and φ angles also indicate a more linear arrangement of the O atom with the S—S bond compared with the other disulfide bonds. Thus, even in the case of AChE the alignment of a peptide O atom with the S—S bond makes it more susceptible to radiation damage.

### 3.6. S⋯O interactions in other proteins and differences in features between the S atoms in susceptible disulfide bonds   

Other proteins have also been reported to have disulfide bonds exhibiting susceptibilities to radiation damage and we consider them next. Winged-bean chymotrypsin inhibitor (WCI) has two disulfide bonds which show damage (Ravelli & McSweeney, 2000 ▸). One of them, Cys135–Cys144, has the polar angles expected for a susceptible disulfide bond (Table 5 ▸). The other, Cys41–Cys85, has a contact between the S and O atoms of Cys41 with a φ angle close to the optimum (−60°), although there is some deviation in θ. Interestingly, however, not only is there a contact between the two atoms of the same residue, as seen in the susceptible disulfide bond in elastase (Table 2 ▸), but the other cysteine residue, Cys85, is also involved in another contact of the same type. Thaumatin is another protein in which the Cys159–Cys164 disulfide bond exhibits an interesting feature (Schulze-Briese *et al.*, 2005 ▸). It occurs in two distinct conformations, which disappear after irradiation. The data in Table 5 ▸ indicate that both the conformations have an S⋯O contact involving the atoms of the same residue (Cys164): conformation 1 has θ, φ angles close to 90°, −60°, whereas conformation 2 has a φ value close to −60° but has an altered θ angle. It has also been proposed that one of the inter-chain disulfide bonds, A:Cys7–B:Cys7, in insulin is sensitive to radiation, whereas the other (A:Cys20–B:Cys19) does not show a significant change after irradiation (Schulze-Briese *et al.*, 2005 ▸). The spherical polar angles given in Table 5 ▸, however, are not able to distinguish between them.

There has also been a noteworthy observation involving the electron density of the S atoms in the susceptible disulfide bonds, one of which seems to retain some residual density, whereas the other appears to becomes detached, sometimes even appearing at a new position with reduced density (Ravelli & McSweeney, 2000 ▸). For the Cys135–Cys144 disulfide bond in WCI, Cys144 has been reported to become detached, and it is remarkable that it is the other cysteine (Cys135) which has the short contact with the carbonyl O atom. (The other disulfide bond is slightly anomalous in the sense that both of the S atoms make contact with O atoms). Thus, it appears that the cysteine which is involved in S⋯O interaction acts as the ‘anchor’ and the other cysteine residue becomes ‘detached’. This agrees with the *ab initio* calculation (Section 3.7), which indicates that the electron flows from the carbonyl O atom to the proximal S atom and then down to the distal S atom, polarizing the S—S bond. Likewise, for the most susceptible Cys6–Cys127 disulfide bond in HEWL, Cys127 is in contact with an O atom (Table 4 ▸) and it is the other cysteine, Cys6, that becomes detached (Ravelli & McSweeney, 2000 ▸). The same feature is observed for the most susceptible Cys254–Cys265 disulfide bond in AChE, where Cys265, which does not have any close O atom contact, becomes detached. Along the same lines, it is also interesting to see that thaumatin, with two conformations of the Cys159–Cys164 disulfide bond, has alternative positions of the Cys159 S atom, while Cys164 with the S⋯O contact behaves as the anchor residue (Table 5 ▸). The susceptible disulfide bond in insulin (A:Cys7–B:Cys7) has also been found to show an asymmetry in the loss of electron density of the two S atoms, with B:Cys7 losing more (Schulze-Briese *et al.*, 2005 ▸). The data presented in Table 5 ▸ are not very clear, with both the atoms having a nearby O atom, but it can be seen that the interaction for A:Cys7 is more linear along the posterior of the S—S bond (θ is 24.5° away from 90°; the value is 53.8° for B:Cys7), making B:Cys7 become detached.

### 3.7. *Ab initio* calculations on S⋯O interaction   

The disulfide–amide model, representing the disulfide and the peptide carbonyl moieties, was constructed. It mimics the ideal values of θ and φ (90° and −60°, respectively) for the optimum inter­action between the Cys30–Cys46 disulfide bond and its closest carbonyl O atom (Fig. 2 ▸). Other interacting geometries, corresponding to different grid values of θ and φ, were also generated, and the potential energy of the system and the charges on the two S atoms were computed for each geometry (Table 6 ▸ using the DFT technique and Supplementary Table S1 using Hartree–Fock theory). The results obtained using both of the techniques indicate that the configuration defined by θ = 90° and φ = −60° has the lowest total energy. At this position the distal S atom has the maximum negative charge, indicating an appreciable change in the polarization of charges over the disulfide bond brought about by the incipient reaction between the nucleophilic O atom and the disulfide group. Reducing the S⋯O distance from 3.08 to 2.9 Å enhances the negative charge on the S atom, whereas increasing the distance (to 3.2 Å) reduces the negative value.

To see whether the orientation of the orbitals on the carbonyl O atom relative to the disulfide plane has any effect on the charge separation between the two S atoms, the CH_3_-CO-NH-CH_3_ molecule was rotated about the C=O axis, keeping the other fragment (Fig. 2 ▸) fixed. The maximum charge separation was found to occur at positions 1, 2, 12 and 13 (Supplementary Table S2), which are in the neighborhood of those found in the crystal structure of elastase.

### 3.8. Natural bond order (NBO) analysis   

**Figure 5:**
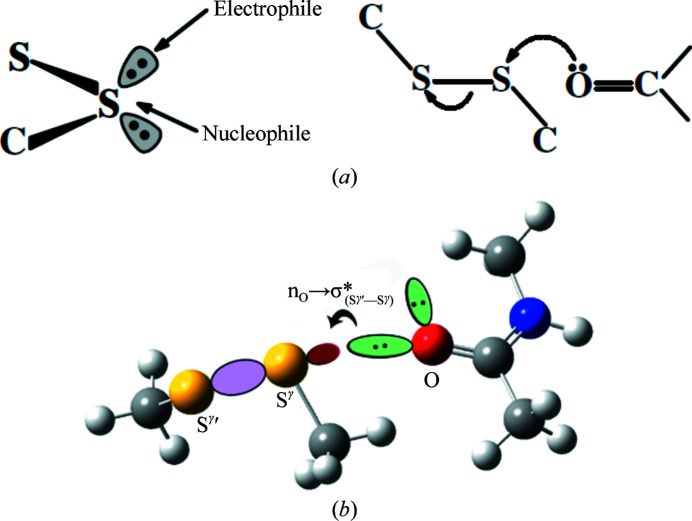
(*a*) The preferred directions of interaction of an electrophile and a nucleophile with respect to the disulfide plane. (An electrophile can also interact with the other lone-pair orbital.) (*b*) The incipient reaction of a nucleophile (the carbonyl group) with the disulfide group. (*c*) Schematic representation of the orbital interaction [n_O_→σ*_(Sγ—Sγ′)_] revealed by NBO calculations.

The second-order perturbation theory analysis in the Fock matrix helps us to understand the delocalization of the electron density from occupied Lewis-type (donor) NBOs to unoccupied non-Lewis-type (acceptor) NBOs, augmenting the analysis of intramolecular and intermolecular interactions and charge transfer in molecular systems (Levine, 1991 ▸). The overlap between the lone electron pair, n, and the vacant antibonding orbital, σ*, causes a change in energy of the lower occupied orbital. This change in energy is referred to as the ‘stabilization energy’ of electron delocalization [*E*(2)]. For each donor NBO (*i*) and acceptor NBO (*j*), the analysis provides the stabilization energy *E*(2), which is estimated as
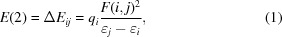
where *q_i_* is the donor-orbital occupancy, ∊*_i_* and ∊*_j_* are diagonal elements and *F*(*i*, *j*) are the off-diagonal NBO Fock matrix elements. If it is zero, there is zero probability of a transition between these two states. Here, (∊_*j*_ − ∊_*i*_) indicates the energy difference between the non-Lewis NBO (*i.e.* σ*) and the orbital occupied by the lone pair, n. Therefore, the *E*(2) value becomes insignificant when the difference becomes zero. A larger *E*(2) value indicates a more donating tendency from electron donors to electron acceptors. Full NBO analysis and second-order Fock matrix perturbation theory analysis was carried out for position 1 (Supplementary Table S2), shown in Fig. 2 ▸, using both DFT/B3LYP/6-31G++(2d,2p) and HF/6-31G++(2d,2p) levels of theory. In addition to the distance in the crystal structure (3.08 Å), two other neighboring distances (2.9 and 3.2 Å) were also used. The perturbation energies of donor–acceptor interactions, as obtained by the two methods, are presented in Table 7 ▸ and Supplementary Table S3. At the S⋯O distance of 3.08 Å the carbonyl O atom (O) has a tendency to transfer its lone-pair electron to the σ* orbital of the S^γ^—S^γ^′ bond [n_O_→σ*_(Sγ—Sγ′)_; Fig. 5 ▸(*c*)] with *E*(2) = 0.54 kcal mol^−1^ (using DFT) and 0.62 kcal mol^−1^ (using Hartree–Fock theory). The values increase to 1.21 and 1.42 kcal mol^−1^, respectively, when the S⋯O distance is reduced to 2.9 Å. In contrast, on increasing the distance to 3.2 Å the *E*(2) values are reduced. This hyperconjugative interaction enhances the negative charge on the distant S atom, showing a similar distance dependence (Table 6 ▸ and Supplementary Table S1), making the S^γ^—S^γ^′ bond susceptible to radiation damage.

## 4. Discussion   

An analysis of the structure of porcine pancreatic elastase based on data sets collected after increasing the duration of radiation exposure shows systematic changes in some bonded and nonbonded parameters. The two most susceptible disulfide bonds show the maximum changes; those for the Cys30–Cys46 disulfide bond are slightly on the higher side. The bond and its oxygen neighbor are shown in Fig. 3 ▸(*b*). For the sensitive disulfide bonds the S—S length increases and, assuming that the bond is between residues *i* and *j*, the angle (C^β^)_*i*_—(S^γ^)_*i*_—(S^γ^′)_*j*_ tends to increase, while the angle (C^β^)_*j*_—(S^γ^)_*j*_—(S^γ^′)_*i*_ decreases (Table 1 ▸). These disulfide bonds also have a carbonyl O atom contacting one of the S atoms along the extension of the S^γ^′—S^γ^ bond (Table 2 ▸, Fig. 4 ▸). The preferred directions of electrophilic and nucleophilic attack on divalent sulfur (Rosenfield *et al.*, 1977 ▸) are shown in Fig. 5 ▸(*a*). The location of an O atom at the back side of the S^γ^′—S^γ^ bond suggests that in these cases the S⋯O interaction represents the incipient state of the reaction of a nucleophile with the disulfide bond [Fig. 5 ▸(*b*)]. On exposure to radiation the reaction proceeds to a different level of completion, weakening the S—S bond. There is also a concomitant increase in the S⋯O distance (Table 3 ▸), indicating a weakening of the nonbonded interaction as the S—S bond is progressively cleaved. Indeed, the geometrical arrangement that gives rise to the electrophile–nucleophile interaction that is observed in small molecules and macromolecules (Bhattacharyya *et al.*, 2004 ▸; Pal & Chakrabarti, 1998 ▸, 2001 ▸; Rosenfield *et al.*, 1977 ▸) is relevant with respect to the intact disulfide moiety. As the S—S bond breaks, the molecular orbitals no longer overlap, thereby increasing the distance between the S and O atoms. The cleavage of the disulfide bond and the occurrence of an alternative rotamer conformation of the resulting free cysteine residues have been observed in the crystal structure (Weik *et al.*, 2000 ▸). The link between the proneness to cleavage of the disulfide bond and the location of a carbonyl O atom along the extension of the S—S bond is further corroborated by the structures of HEWL, AChE and other proteins, in which the most sensitive disulfide bridge also exhibits a similar atomic arrangement [Tables 4 ▸ and 5 ▸, Fig. 4 ▸(*b*)].

Upon irradiation, the disulfide bond (*R*SS*R*) forms a radical anion (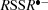) that might subsequently be protonated (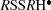); this dissociates into a thiol (*R*SH) and a thiyl radical (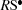) (Weik *et al.*, 2002 ▸). There is no evidence of any direct correlation between the solvent accessibility of the cysteine residues and their inclination towards radiation damage (Carugo & Carugo, 2005 ▸). There are semi-quantitative indications that flexible regions are more affected than those that are located in helices or strands (Weik *et al.*, 2001 ▸). Here, we have demonstrated how a stabilizing electrophile–nucleophile interaction (Bhattacharyya *et al.*, 2004 ▸; Pal & Chakrabarti, 1998 ▸, 2001 ▸; Rosenfield *et al.*, 1977 ▸) formed by the placement of a main-chain O atom along the S—S bond extension can provide a pathway for the transfer of an electron to the disulfide bond (Fig. 5 ▸) (which makes the distal S atom carry more negative charge; Table 6 ▸), leading to its break up and thereby providing an explanation of the difference in the susceptibilities of disulfide bonds to radiation damage. This also rationalizes the observation that one of the S atoms retains some residual density, while the other moves away (Ravelli & McSweeney, 2000 ▸), with the former being that with the S⋯O contact, as revealed in our analysis. It has been suggested that disulfide bonds and metal sites in structures deposited in the Protein Data Bank (Berman *et al.*, 2000 ▸) might be severely affected by the exposure of protein crystals to X-rays and require reinvestigation (Ravelli & McSweeney, 2000 ▸). An analysis of the environment of the disulfide bonds would be a way to identify structures that might be harboring the specific interactions that would make a disulfide bond susceptible to radiation-induced cleavage, and these would then be candidates for further study. In a related study, the chemical reactivity of disulfide bonds has also been shown to be influenced by their structure and environment (Karimi *et al.*, 2016 ▸).

The S⋯O interaction is a wider manifestation of what has been observed in small molecules: short contacts involving a nucleophile and the carbonyl C atom (Bürgi *et al.*, 1973 ▸). A nucleophile donates lone-pair (n) electron density into the empty π* orbital of a carbonyl group. The carbonyl–carbonyl interaction, where the carbonyl O atom is the nucleophile, is rather abundant in protein structures (Fufezan, 2010 ▸; Newberry & Raines, 2017 ▸). A telltale sign of the S⋯O interaction has recently been provided by the ω-turn. In this β-turn mimic, the C^γ^–H group in the side chain forms a C—H⋯O hydrogen bond with the main-chain carbonyl group two residues ahead of it (Dhar *et al.*, 2015 ▸). The residue with the highest propensity to occur at this position is methionine, as along with the C—H⋯O interaction the side-chain S atom can simultaneously be involved in another S⋯O interaction, stabilizing the motif. Here, we find that when present involving a disulfide bond, the normally stabilizing S⋯O interaction allows the interacting groups to move along the reaction trajectory under X-ray exposure, thus becoming deleterious to the integrity of the disulfide bond. This is a unique example in protein structures where a normally stabilizing interaction appears to be responsible for cleavage of the disulfide bond when the crystal is exposed to intense X-ray radiation.

## 5. Conclusions   

Disulfide-bond cleavage has been reported under conditions of synchrotron radiation. There appears to be a correspondence between the inclination of the disulfide bond to break and the location of a carbonyl O atom along the extension of the S—S bond, providing a pathway for electron transfer for reduction of the bond. The study exemplifies the importance of weak interactions other than hydrogen bonding in controlling the stability of protein structures.

## Supplementary Material

Supplementary Tables. DOI: 10.1107/S2052252520008520/lz5035sup1.pdf

## Figures and Tables

**Table:** S^γ^—S^γ^′ bond length (Å).

	Data set												
S—S bond	A-0	A-1	A-2	A-3	A-4	A-5	A-6	A-7	A-8	A-9	A-10	A-11	A-12
158–174	2.07	2.07	2.07	2.08	2.08	2.08	2.08	2.08	2.07	2.09	2.07	2.08	2.08
30–46	2.03	2.06	2.07	2.08	2.09	2.09	2.09	2.10	2.10	2.10	2.09	2.09	2.09
127–194	2.01	2.02	2.03	2.03	2.04	2.04	2.04	2.04	2.05	2.05	2.06	2.05	2.06
184–214	2.02	2.02	2.02	2.02	2.03	2.03	2.03	2.03	2.03	2.03	2.03	2.04	2.04

**Table:** C^β^—S^γ^—S^γ^′ bond angle (°). The two entries correspond to the two angles centered on the two S atoms (given in the same order as the residue numbers).

	Data set												
S—S bond	A-0	A-1	A-2	A-3	A-4	A-5	A-6	A-7	A-8	A-9	A-10	A-11	A-12
158–174	103.7, 98.5	103.7, 99.0	103.9, 98.6	104.0, 98.2	104.9, 98.1	105.1, 97.8	105.0, 98.3	105.1, 97.7	105.2, 97.4	105.3, 97.6	106.2, 97.2	106.4, 97.8	106.7, 97.7
30–46	103.7, 100.9	104.0, 99.5	103.3, 98.9	103.8, 98.5	104.1, 97.8	104.1, 98.0	104.7, 97.7	104.8, 97.0	105.4, 96.9	105.6, 97.0	105.4, 96.9	106.6, 96.5	106.5, 96.4
127–194	106.1, 102.1	107.1, 101.9	107.0, 102.7	107.1, 102.8	106.7, 102.2	106.8, 102.8	106.5, 102.6	108.4, 102.2	107.3, 101.9	107.8, 103.5	107.0, 102.1	108.3, 102.2	108.4, 102.1
184–214	107.2, 104.0	107.3, 103.8	107.5, 103.5	107.9, 104.3	107.6, 103.8	107.4, 104.0	107.5, 104.2	107.0, 104.2	107.4, 103.9	107.5, 104.4	107.4, 103.9	106.7, 103.8	106.6, 103.8

**Table:** The torsion angles χ_3_, χ_1_, χ_2_, χ_1′_ and χ_2′_ (°) do not show any noticeable change with exposure. The torsion angles from the first data set (A-0) are given below.

S—S bond	χ_3_	χ_1_	χ_2_	χ_1′_	χ_2′_
158–174	−89.9	−160.0	−179.3	−57.0	−171.4
30–46	−87.1	−84.2	−150.5	−69.6	−90.5
127–194	103.3	−59.2	−102.4	−55.0	−89.7
184–214	71.1	−156.3	43.2	−65.0	173.4

**Table 2:** Nonbonded S⋯O contacts in elastase Corresponding to the first data set (A-0). The S—S bonds are arranged in decreasing order of susceptibility.

	Contacts		Parameters				
S—S bond	S	O†	S⋯O (Å)	C^β^—S^γ^—O (°)	S^γ^′—S^γ^—O (°)	θ (°)	φ (°)
158–174	158	C158	3.24	72.0	147.7	58	−59
30–46	30	T29	3.13	80.6	172.6	84	−48
		S188	3.36	65.7	75.8	33	−169
	46	A43	3.95	56.4	156.8	86	−73
		S188	3.47	147.0	69.6	59	63
127–194	127	S125	3.67	98.2	152.0	76	−28
		P126	3.33	80.6	104.6	16	−74
	194	H193	3.73	77.7	88.0	13	−150
184–214	184	L134	4.00	50.7	151.7	74	−78
		R136	3.73	122.8	124.1	68	−1
	214	G183	3.82	57.8	86.6	35	−148

† The residue number preceded by the one-letter amino-acid code of the residue providing the main-chain carbonyl O atom.

**Table 3:** Changes in the S⋯O distance (Å) in elastase

	Contact		Data set												
S—S bond	S	O†	A-0	A-1	A-2	A-3	A-4	A-5	A-6	A-7	A-8	A-9	A-10	A-11	A-12
158–174	158	C158	3.24	3.25	3.27	3.26	3.28	3.31	3.29	3.28	3.29	3.30	3.31	3.32	3.33
30–46	30	T29	3.13	3.16	3.18	3.21	3.24	3.28	3.30	3.31	3.34	3.36	3.34	3.34	3.35
		S188	3.36	3.37	3.37	3.4	3.39	3.37	3.40	3.43	3.38	3.40	3.39	3.41	3.41
	46	A43	3.95	3.94	3.92	3.92	3.94	3.92	3.94	3.97	3.95	3.94	3.94	3.94	3.93
		S188	3.47	3.46	3.45	3.47	3.48	3.46	3.48	3.49	3.48	3.49	3.47	3.49	3.49
127–194	127	S125	3.67	3.71	3.71	3.71	3.70	3.72	3.73	3.74	3.72	3.71	3.74	3.74	3.75
		P126	3.33	3.35	3.31	3.34	3.35	3.37	3.34	3.31	3.35	3.33	3.35	3.37	3.37
	194	H193	3.73	3.74	3.73	3.71	3.72	3.75	3.72	3.74	3.72	3.71	3.71	3.74	3.74
184–214	184	L134‡	4.00						4.00						
		R136	3.73	3.71	3.75	3.74	3.75	3.76	3.76	3.80	3.81	3.81	3.81	3.81	3.82
	214	G183	3.82	3.83	3.81	3.83	3.84	3.83	3.85	3.84	3.86	3.85	3.86	3.86	3.87

† The residue number preceded by the one-letter amino-acid code of the residue providing the O atom is given. Some S atoms can interact with two O atoms.

‡ Only distances of ≤4.0 Å are shown.

**Table 4:** Nonbonded S⋯O contacts in HEWL and AChE The first entry in both cases is susceptible to radiation damage; the second entry for HEWL is partially susceptible.

	Contact		Parameter				
S—S bond†	S	O‡	S⋯O (Å)	C^β^—S^γ^—O (°)	S^γ^′—S^γ^—O (°)	θ (°)	φ (°)
HEWL							
6–127	127	I124	3.40	75	140	50	−57
76–94	94	C94	3.51	69	83	25	−159
30–115	115	C30	3.51	158	73	69	57
64–80	80	C64	3.46	159	75	69	53
AChE							
254–265	254	G249	3.68	65	146	59	−68
67–94	94	C94	3.54	53	84	41	−151
402–521	521	R517	3.27	81	120	30	−56

† The relative accessibilities of the cysteine residue pairs are as follows: HEWL, (33.4, 16.6), (11.5, 1.4), (0.6, 0) and (0.1, 1.1); AChE, (10.2, 15.8), (3.5, 1.1) and (0, 6.3).

‡ The main-chain O atom which is in shortest contact with the disulfide bond is considered. Its one-letter amino-acid code and the residue number are indicated.

**Table 5:** Nonbonded S⋯O contacts present in additional proteins

	Contact		Parameter				
S—S bond	S	O†	S⋯O (Å)	C^β^—S^γ^—O (°)	S^γ^′—S^γ^—O (°)	θ (°)	φ (°)
WCI							
41–85	41	A:C41	3.62	69.5	118.7	33.2	−79
	85	A:C85	3.60	57.9	109.0	35.3	−106
135–144	135	A:V116	3.64	78.8	176.8	86.9	−51
Thaumatin							
159–164 (conformation 1)	164	A:C164	3.36	73.0	165.5	76.9	−50.6
159–164 (conformation 2)	164	A:C164	3.36	73.0	124.9	35.9	−66.0
Insulin (inter-chain disulfide bonds)							
A7–B7	A7	A:V3	3.78	57.3	149.2	65.5	−72.8
	B7	B:C7	3.40	73.9	124.6	36.2	−66.9
A20–B19	A20	A:E17	3.42	78.3	146.1	56.2	−51.7
A20–B19	B19	B:L15	3.18	72.7	138.0	48.7	−60.9
	B19	B:G23	3.71	65.8	101.5	24.9	−114.3

† The chain identifier is provided before the one-letter amino-acid code and residue number.

**Table 6:** Charges on the S atom and the total energy of the system [as calculated using DFT theory with the B3LYP functional and basis set 6-­31++G(2d,2p)] at different values of θ and φ and an S⋯O distance of 3.08 Å The charges on distant and neighboring S atoms (S^γ^′ and S^γ^, respectively) are given in parentheses. To calculate the total energy, *E*_RHF_ (in atomic units) obtained from the program was first converted into kcal mol^−1^. The value at a given (θ, φ) was then expressed relative to that at (90°, −60°), *i.e.* Δ*E* = *E*_RHF(θ, φ)_ − *E*_RHF(__90°, −60°)_. Calculations were also performed at two distances at either side of 3.08 Å and the resulting values are (−0.173, 0.039) at 2.9 Å and (−0.144, 0.017) at 3.2 Å.

	θ (°)		
φ (°)	90	45	0
−60	0 (−0.155, 0.026)	0.88 (−0.097, 0.002)	4.33 (0.224, −0.215)
0	0.88 (−0.025, 0.069)	3.33 (−0.120, 0.042)	
+50	0.94 (−0.069, −0.007)	1.94 (0.068, −0.096)	

**Table 7:** Second-order perturbation theory analysis of the Fock matrix on an NBO basis (using DFT theory) of the model shown in Fig. 2 ▸ representing elastase

S^γ^⋯O distance (Å)	Donor (*i*)	Type	Acceptor (*j*)	Type	*E*(2)† (kcal mol^−1^)	∊(*j*) − ∊(*i*)‡ (atomic units)
2.9	O	Lp (1)	S^γ^—S^γ^′	σ*	1.21	0.80
		Lp (2)			0.17	0.34
3.08	O	Lp (1)	S^γ^—S^γ^′	σ*	0.54	0.79
		Lp (2)			0.08	0.33
3.2	O	Lp (1)	S^γ^—S^γ^′	σ*	0.32	0.79
		Lp (2)			0.05	0.33

† *E*(2) is the energy of hyperconjugative interaction (stabilization energy). The default threshold of 0.05 kcal mol^−1^ was used.

‡ The energy difference between donor (*i*) and acceptor (*j*) NBO orbitals.
