# Supplementary material for: The susceptibility of disulfide bonds towards radiation damage may be explained by S⋯O interactions
Source: IUCrJ. 2020 Jul 24;7(Pt 5):825–34. doi: 10.1107/S2052252520008520 (PMC7467163; doi:10.1107/S2052252520008520)
Supplement: Supplementary file 1 [file m-07-00825-sup1.pdf]

IUCrJ

Volume 7 (2020)

Supporting information for article:

The susceptibility of disulfide bonds towards radiation damage may be explained by S⋯O interactions

Rajasri Bhattacharyya, Jesmita Dhar, Shubhra Ghosh Dastidar, Pinak Chakrabarti and Manfred S. Weiss

Table S1 Charges[a] on the S atom and the total energy[b] of the system (as calculated using Hartree-Fock theory with basis set 6-31++G(2d,2p)) at different values of θ and ϕ, and S···O distance of 3.08 Å[c].

**ϕ (°)**	**θ(°)[a]**		
	**90**	**45**	**0**
**-60[b]**	0.0<br>(-0.297, 0.005)	0.63<br>(-0.187, -0.063)	5.02<br>(-0.233, 0.066)
**0**	0.94<br>(-0.163, -0.084)	3.89<br>(-0.192, 0.038)	
**+50**	0.31<br>(-0.074, -0.070)	2.01<br>(-0.066, -0.158)	

[a] The charges on distant and neighboring S atoms (S_{γ}' and S_{γ}, respectively) are given in parenthesis.

[b] (E_{RHF}) (a.u) obtained from the program was first converted into kcal/mol. The value at a given (θ, ϕ) was then expressed relative to that at (90°, -60°), *i.e.*, $\Delta E = E_{RHF(\theta,\phi)} - E_{RHF(90,-60)}$.

[c] Calculations were also done at two distances on either side of 3.08 Å , and the resulting charges are: (-0.319, 0.014) at 2.9 Å and (-0.284, -0.001) at 3.2 Å.

Table S2 Charges on the S atoms and energy of interaction when the amide group is rotated about the C=O axis, keeping the disulphide moiety fixed (using DFT/B3LYP/6-31G++(2d,2p) level of theory).

Position	Dihedral angle(°)[a]	Charge on distant S atom	Charge on proximal S atom
1	-12	-0.155	0.026
2	-42	-0.154	0.019
3	-72	-0.151	-0.001
4	-102	-0.146	0.001
5	-132	-0.142	-0.011
6	-162	-0.143	-0.002
7	-180(or +180)	-0.139	-0.002
8	+150	-0.138	0.016
9	+120	-0.151	0.013
10	+90	-0.148	0.001
11	+60	-0.151	0.015
12	+30	-0.157	0.031
13	0	-0.157	0.029

[a] The virtual dihedral angle is defined by $S_\gamma \cdots O\text{-}C\text{-}CH_3$. The position 1 corresponds to what is shown in Fig. 2.

Table S3 Second order perturbation theory analysis of the Fock matrix in NBO basis (using Hartree-Fock theory) of the model shown in Fig. 2 representing elastase

$S_\gamma \cdots O$ distance (Å)	Donor (i)	Type	Acceptor (j)	Type	E(2)[a] (kcal/mol)	ε(j)-ε(i)[b] (a.u)
2.9	O	LP (1)	Sγ-Sγ′	σ*	1.42	1.25
		LP (2)			0.18	0.72
3.08	O	LP (1)	Sγ-Sγ′	σ*	0.62	1.25
		LP (2)			0.08	0.71
3.2	O	LP (1)	Sγ-Sγ′	σ*	0.35	1.25
		LP (2)			0.05	0.71

[a] E(2) means energy of hyperconjugative interaction (stabilization energy). The default threshold of 0.05 kcal/mol was used.

[b] Energy difference between donor (i) and acceptor (j) NBO orbitals.
